# Longitudinal change in the diet's monetary value is associated with its change in quality and micronutrient adequacy among urban adults

**DOI:** 10.1371/journal.pone.0204141

**Published:** 2018-10-12

**Authors:** May A. Beydoun, Marie T. Fanelli-Kuczmarski, Jennifer Poti, Allyssa Allen, Hind A. Beydoun, Michele K. Evans, Alan B. Zonderman

**Affiliations:** 1 Laboratory of Epidemiology and Population Sciences, National Institute on Aging, Intramural Research Program, NIA/NIH/IRP, Baltimore, MD, United States of America; 2 Department of Behavioral Health and Nutrition, University of Delaware, Newark, DE, United States of America; 3 Department of Nutrition and Carolina Population Center, University of North Carolina at Chapel Hill, Chapel Hill, NC, United States of America; 4 Centers for Medicare and Medicaid Services (CMS), Woodlawn, Maryland, United States of America; 5 Department of Medicine, Johns Hopkins Medical Institutions, Johns Hopkins University, Baltimore, MD, United States of America; Universidade de Sao Paulo, BRAZIL

## Abstract

**Background:**

Reducing diet costs may lead to the selection of energy-dense foods, such as refined grains or foods high in added sugars and/or fats, which can lower overall dietary quality. We examined the longitudinal association between the monetary value of the diet (MVD) and the overall dietary quality across sex, race and income groups.

**Methods and findings:**

Longitudinal data from 1,466 adult urban participants from Healthy Aging in Neighborhoods of Diversity across the Life Span (HANDLS) study were used. Healthy Eating Index–2010 (HEI–2010) and Mean Adequacy Ratio (MAR) were computed and a national food price database was used to estimate MVD. Multiple linear regression analyses were conducted linking annual rates of change (Δ) in MVD to ΔHEI-2010 and ΔMAR, stratifying by sex, race and income groups. Among key findings, ΔHEI-2010 was comparable across socio-demographic groups, while ΔMAR was higher among women and individuals above poverty. Adjusting for key covariates, ΔMVD was positively associated with both ΔHEI-2010 and ΔMAR, and with a consistently stronger association among individuals above poverty, specifically for the total proteins and empty calories components of HEI-2010 and several nutrient adequacy ratios (NARs: vitamins C, E, B-6 and Zinc). ΔMVD-ΔMAR association was stronger in women, mainly influenced by ΔMVD’s positive associations with B-vitamins, copper, calcium, magnesium and phosphorus NARs. ΔMVD-Δvitamin D NAR’s positive relationship was stronger among Whites, while ΔMVD-Δvitamin B-12 NAR’s association was stronger among African-Americans.

**Conclusions:**

In sum, a potential increase in MVD may have a stronger impact on dietary quality among urban adult women and above-poverty individuals.

## Introduction

Evaluating the healthfulness of dietary patterns is a key challenge when studying diet-disease relationships. The US federal government established a set of recommendations for an optimal quality of the total diet through providing Dietary Guidelines for Americans (DGA), while setting some benchmarks to would lead people closer towards these specific recommendations through the Healthy People objectives.[1] Those objectives are outlined in the Health People 2020 and the Dietary Guidelines for Americans.[2,3] The latter provides evidence-based information on healthfulness of diets.[4]Thus, the total diet quality is often measured with various indices relying on combinations of food groups and nutrients. Among those that are non-data driven, the Healthy Eating Index-2010 (HEI-2010) is an energy-adjusted food-group based index evaluating people’s conformity to most recent Dietary Guidelines for Americans. [5–9] Similarly, the Mean Adequacy Ratio (MAR) aims at comparing an individual’s mean micronutrient intake to its Recommended Dietary Allowance (RDA).[10] Importantly, food choice determinants, particularly those of economic nature, must be examined.

Altering food choice can modify diet quality to be consistent with recent guidelines. Those foods choices strongly depend on people’s health status, preferences, cultural influences and more importantly socio-economic constraints, including income. Under financial constraints, food expenditure experiences substantial cuts relative to other portions of household expenditures, resulting in the choice of less expensive foods.[11] Reducing diet costs is often accomplished by selecting more energy-dense foods, such as refined grains or foods high in added sugars and/or fats, resulting in lower overall dietary quality.[12–16]

While current research focuses on associations between food prices at the regional, city and neighborhood levels and food consumption behaviors and patterns, [17–22] examining the monetary value of the diet (MVD) and how it is related to various dietary components and quality has gained more recent interest. [10,23–53] Most studies reported a direct relationship between MVD and diet quality (or an inverse relationship with consumption of energy-dense foods),[10,23–31,33–38,40–42,44–47,50,51,54–60], a finding not replicated in other studies.[32,39,43,48,49]

To our knowledge, this study is the first to examine the net association between annual rates of change in MVD and dietary quality, across sex, race and poverty status, using data from a large sample of low-income urban US adults, the Healthy Aging in Neighborhoods of Diversity across the Life Span (HANDLS).

## Methods

### Database

HANDLS is a prospective cohort study that is ongoing since 2004. The study primarily focuses on disparities in cardiovascular and cognitive health of a socioeconomically diverse sample of Whites and African-Americans (30–64 years old at baseline), representing selected neighborhoods in Baltimore, Maryland. While the HANDLS sample selection methodology is described in greater detail elsewhere [61], in brief, it uses an area probability sampling strategy of thirteen neighborhoods. Phase 1 of the baseline visit (also known as (aka) visit 1 (in our study) or Wave 1 (in HANDLS): 2004–2009) consisted of screening, recruitment, first dietary recall, and household interviews, whereas phase 2 of visit 1 consisted of the second dietary recall and examinations in mobile Medical Research Vehicles (MRV). Two 24-hr recalls were also collected at the follow-up visit [aka visit 2 (in our study) or wave 3 (in HANDLS): 2009–2013]. Longitudinal data from participants examined both during the baseline visit 1 (2004–2009) and the first follow-up visit 2 (2009–2013) were used in this study, with a mean follow-up time±SE of 4.62y±0.95 (range: 0.42–8.20).

Written informed consent was obtained from all participants who accessed a protocol booklet in layman's terms and a video that described all procedures and future re-contacts. HANDLS study was ethically approved by the National Institutes of Health, National Institute of Environmental Health Sciences (NIEHS/NIH) Institutional Review Board.

### Study sample

Of the original HANDLS sample selected at wave 1 (N = 3,720), 2,177 had complete data on two 24-hour dietary recalls at baseline collected both at Phase 1 (household visit), and Phase 2 (MRV visit). Out of those, we excluded participants with incomplete dietary recalls from the first follow-up visit, resulting in a sample of N = 1,516. An additional 50 individuals had missing data on several covariates including literacy, education, self-rated health and body mass index. Missing data on smoking/drug use were included as a dummy variable in multivariable models. This resulted in a final sample of N = 1,466 (**S1 Fig**). Compared with the sample with complete baseline dietary data (N = 2,177), the final selected sample with complete data on both waves and covariates (N = 1,466) differed from those excluded, by having a higher proportion of women, African-Americans and individuals above poverty. This sample selectivity was adjusted for using a 2-stage Heckman selection model (See statistical analysis section).

### Dietary assessment

At each of two HANDLS visits (i.e. visits 1 and 2), two 24-hour dietary recalls were collected through the utilization of the US Department of Agriculture (USDA) Automated Multiple Pass Method, a well-established computerized structured interview.[62] Several measurement aids were used, including measuring cups, spoons, rulers, and an illustrated Food Model Booklet which allowed participants to report accurate food and beverage quantities that were consumed. Both recalls were administered in-person by trained interviewers, 4 to 10 days apart, during the visit 1 study period (2004–2009) while one of two recalls was administered by a telephone interview during the visit 2 study period (2009–2013). The coding process of the dietary recall was completed by trained nutrition professionals using the Survey Net statistical software,[63] which matched foods consumed with 8-digit codes identified in the Food and Nutrient Database for Dietary Studies (FNDDS) version 3.0 for baseline visit 1 and version 5 for the follow-up visit 2. [64]

### Key outcome measures

#### Healthy Eating Index– 2010

HEI-2010’s computational steps and statistical code for the 24-hr recalls can be found at the National Cancer Institute website on Applied Research.[65] Moreover, specifically for the HANDLS study, detailed description of the steps used can be found elsewhere. [66] For each recall day (days 1 and 2) and each study visit, total and component HEI-2010 scores were computed. Those estimates were subsequently averaged to obtain the mean HEI-2010 total and component scores for both days combined for each of two visits. HEI-2010 and components were measured at both HANDLS visits (DQ_base_ and DQ_follow_) and annual rate of change (Δ) was measured as: (DQ_follow_-DQ_base_)/(Age_follow_-Age_base_).

#### Mean Adequacy Ratio (MAR) and Nutrient Adequacy Ratio (NAR)

The MAR and NAR nutrient-based diet quality were estimated using methods that were published elsewhere [67,68]. Actual dietary intakes relative to RDAs of selected vitamins and minerals (i.e. calcium, magnesium, phosphorus, Vitamins A, C, D, E, B-6, folate, B-12, iron, thiamin, riboflavin, niacin, copper, and zinc) were used to compute a second diet quality index. From the RDA and actual intake of each vitamin and mineral considered, a nutrient adequacy ratio (NAR) was estimated, as follows: NAR = Subject’s actual daily nutrient intake divided by the RDA of that nutrient. Moreover, 35 mg of vitamin C was added to the RDA of participants reported as being current smokers.[69] Each NAR was then expressed as percentage and truncated at 100%. [68] The mean adequacy ratio (MAR), a nutrient-based measure of overall dietary quality, was computed using as the sum of all 16 nutrient NARs divided by 16. Annual rates of change in MAR and NAR were computed using a similar approach as for HEI-2010 and components.

#### Monetary value of diet estimation (MVD)

Diet cost per 100 grams was estimated using the Global Food Research Program at UNC’s Packaged Food Purchase and Price Database, 2004–13, which provides average national and market-specific prices of ~3,700 foods in “as-purchased” form per quarter. [70,71] Prices are derived from packaged foods including minimally processed foods, commercially prepared ready-to-eat foods and commercially formulated or prepared dishes. The database was generated by linking food and beverage purchase data from the Nielsen Homescan Consumer Panel to Nutrition Facts Panel data from various sources including the Mintel Global New Products Database. [72,73] Detailed design of the Homescan study is provided in **S1 Method**. Using descriptions and ingredient lists, products were categorized at the barcode-level into 34 food and 8 beverage groups based on nutritional content and consumption patterns; this methodology and the UNC Homescan food groups were previously described.[74] Market-specific price per 100 g for each food group in each quarter was calculated by dividing the survey-weighted dollars spent by weighted volume of purchases for all products in a given food group by households in that market during the quarter. [70] For this study, average real prices of each food group were calculated for all households in the Baltimore market and scaled to the first quarter of 2004 to account for inflation and allow comparability over time. All 8-digit codes in the Food and Nutrient Database for Dietary Studies (FNDDS) versions 4.1, 5.0 and 2011–12 reported by National Health and Nutrition Examination Survey participants from stores or vending in 2007–08 to 2011–12 were matched to UNC Homescan food groups. This database provided food prices per 100g at the national level for about 3,700 FNDDS food codes, determined from 1,934,441 barcoded products (814,481 unique Nutrition Facts Panel records), and facilitated MVD per day estimates among HANDLS participants.

Given limited linkage between Homescan food prices with NHANES 2007–08 and 2011–12, half of the remaining FNDDS food codes reported by HANDLS participants were imputed, by matching previously computed food groups for HANDLS (60 food and beverage groups) with 42 food and beverage groups computed by UNC. When a reasonable match was not found, a nearest neighbor code matched to one of the 42 groups was used for imputation. This resulted in a harmonization algorithm by which all reported HANDLS FNDDS food codes were placed in one of 42 food groups and then linked to a singular food price, given the year and quarter in which they were reported. Taking the example of wave 1, day 1, it is shown that some food groups rank high in terms of prevalence of reporting for both the imputed and non-imputed observations, starting from individual food codes. This validates the imputation technique through the harmonization of food groups. (**S2 Method**).

Total cost was measured by adding food prices within individual HANDLS ID, given the amount consumed per food code per recall and individual. The MVD was then estimated per individual per wave as average MVD across recalls for each wave. Annual rate of change in MVD was estimated as follows: ΔMVD = (MVD_follow_-MVD_base_)/(Age_follow_-Age_base_).

### Covariates

Several baseline or fixed covariates were considered as potential confounders and/or effect modifiers. Those included age, sex, race (White *vs*. African American), completed years of education (<High School (HS); HS and >HS), literacy (WRAT-3 total score), poverty status, a design-based binary variable in HANDLS based on poverty income ratio (PIR<125%: below poverty; PIR≥125%: above poverty), current smoking status (0: “never or former smoker” and 1 “current smoker”) and current drug use (0:”never or former drug user and 1 “current drug user”). The reading subtest of the Wide Range Achievement Test-3^rd^ Edition (WRAT-3), a widely validated measure of literacy, assessed participants’ ability to recognize and name letters and words, with a total score computed as “total correctly pronounced letters + total correctly pronounced words”.[75] Other baseline covariates included employment status, body mass index (weight/squared-height, kg.m^-2^) and self-rated health (0: Poor/fair, 1: Good, 2: Very good/excellent). Finally, models were also adjusted for % energy consumed at home (food items purchased at grocery stores) to account for price inflation in away-from-home settings. In fact, since most of the MVDs were based on estimated prices of foods as sold in grocery stores and prepared at home rather than away from home food prices, it was important to adjust for the % energy consumed at home in all our models. Energy intake (kcal/d) from the total diet was adjusted for in all regression models. In both latter cases, annual rates of change (Δ) were entered rather than baseline values, given the strong association between energy intake and MVD.

### Statistical analysis

Stata release 14.0 (StataCorp, College Station, TX) was used to complete all statistical analyses.[76] First, study sample characteristics were assessed by tertiles of annual rates of change in MVD (ΔMVD tertile), as was done in our previous cross-sectional analyses.[10] To test linear trend relationship between MVD tertiles and continuous characteristics, a bivariate ordinary least square (OLS) regression was used with MVD entered as an ordinal predictor of each continuous variable of interest. Associations between categorical study characteristics and MVD tertiles were evaluated with χ^2^ tests. Second, means of baseline, follow-up and annual rates of change in diet quality indices (total scores on HEI-2010, MAR and individual components) were compared across sex, race and poverty status groups using independent samples *t*-tests. Finally, multiple OLS regression models were conducted to examine the net association between annual rates of change in MVD and the annual rate of change in different diet quality indices, adjusting for potentially confounding factors and stratifying by sex, race and poverty status. To test statistically the moderation by those socio-demographic factors separately, interaction terms were added to the unstratified multivariable models (i.e. ΔMVD×sex, ΔMVD×race and ΔMVD×pov), while also including the main effects and the other covariates. Predictive margins of annual rates of change in HEI-2010 total score and MAR were plotted from those models, against MVD tertiles and across each of the three socio-demographic moderating factors.

The non-random selection of participants with complete data from the target study population can often lead to selection bias. To account for this type of bias, a 2-stage Heckman selection model was constructed,[77] using a probit model to obtain an inverse Mills ratio at the first stage (derived from the predicted probability of being selected out of the sample with complete 24 hr recalls at baseline (N = 2,177, **S1 Fig**), conditional on the covariates in the probit model, mainly baseline age, sex, race, poverty status and education), as was done in earlier studies.[10,78–80] Specifically, participants selected into the final analytic sample compared to those excluded from the total with complete and adequate baseline dietary data (i.e. N = 1,466 vs. N = 711) were more likely to be women (59.3% vs. 51.1%, P<0.001), African-American (59.8% vs. 54.0%, P = 0.010), and to have above poverty status (58.7% vs. 53.7%, P = 0.027). When comparing the final selected sample (N = 1,466) to those excluded from the original phase I, visit 1 sample (N = 2,254 of 3,720), only sex differentials were observed whereby women were likely to be selected (59.3% vs. 51.7%). Thus, we chose to include the inverse mills ratio that adjusted for selectivity between baseline and follow-up dietary data completeness, given its ability to adjust for greater bias.

Type I error was set at 0.05 for main effects and 0.10 for interaction terms due to the latter’s reduced statistical power compared to the former.[81]

## Results

The distribution of selected fixed, baseline and annual rate of change (Δ) values of study characteristics by ΔMVD tertile are presented in **Table 1**. Overall, both baseline (-) and follow-up (+) energy intakes (total and from grocery stores) were linearly related to ΔMVD (P-trend<0.001), though Δenergy intake (kcal/d/y) and Δ % energy from grocery stores were not related to ΔMVD. While ΔMVD was not associated with race, age or poverty status, percent male was over-represented in the lowest tertile of ΔMVD (45.2% (T_1_) vs. 36.2% (T_2_) and 41.2% (T_3_)). Moreover, a higher proportion rated their health as poor/average when belonging to the lowest tertile ΔMVD, as compared to the middle and uppermost tertiles (T1: 26.4% vs. T2: 19.4% and T3: 23.4%).

**Table 1:** Study sample characteristics by tertile of longitudinal change in the monetary value of diet (ΔMVD, $/d), HANDLS 2004–2013^3^.

	ΔMVD tertiles ($/d/y) ^1^			
	T_1_	T_2_	T_3_	P ^2^
	(N = 489)	(N = 489)	(N = 488)	
Range,$/d/y:	-6.053; -0.168	-0.168; +0.223	+0.223;+2.261	
**Monetary value of diet and energy intakes**				
Monetary value of diet at baseline, $/day (X ± SE)	8.1±0.1	5.6±0.1	5.0±0.1	**<0.001**
Monetary value of diet at follow-up, $/day (X ± SE)	5.2±0.1	5.6±0.1	7.4±0.1	**<0.001**
ΔMVD, $/d/y (X ± SE) ^1^	-0.64±0.02	-0.01±0.01	+0.54±0.01	**<0.001**
Energy intake at baseline, kcal/d (X ± SE)	2,504±49	1,872±33	1,650±34	**<0.001**
Energy intake at follow-up, kcal/d (X ± SE)	1,836±37	1,918±32	2,396±41	**<0.001**
Δ Energy intake, kcal/d/y (X ± SE) ^1^	-152.9±9.8	+10.0±5.6	+169±8	0.11
**Baseline socio-demographic and SES variables**				
Sex, % male	45.2	36.2	41.2	**0.023**
Age at baseline, yrs. (X ± SE)	48.5±0.41	48.7±0.4	48.1±0.4	0.57
Age at follow-up, yrs. (X ± SE)	53.1±0.41	53.5±0.4	52.7±0.4	0.38
Δ Age, yrs. (X ± SE)	4.60±0.04	4.80±0.03	4.60±0.04	**<0.001**
African-American, %	56.9	60.7	60.3	0.75
Poverty status, % (<125% PIR)	40.5	44.2	38.3	0.18
Education, yrs. completed (X ± SE)				0.81
<HS	6.1	7.6	5.9	
HS	58.1	57.5	56.4	
>HS	35.8	35.0	37.5	
Literacy, WRAT-3 score				0.31
<36, %	21.3	23.7	19.3	
37–40, %	16.4	13.3	15.2	
41–46,%	26.6	29.7	29.3	
≥47,%	35.8	33.3	36.3	
% Unemployed in last month, yes	34.0	34.4	32.6	0.51
% Unemployment in last month, missing	19.4	15.1	17.8	
**Baseline drug and tobacco use**				
Any drug, current user, %	17.6	15.5	14.6	0.22
Any drug, missing, %	5.3	5.1	8.0	
Tobacco, current user, %	45.4	41.5	39.6	0.13
Tobacco, missing, %	5.3	5.1	8.0	
Baseline body mass index, kg/m^2^(X ± SE)	30.1±0.3	29.8±0.3	30.1±0.3	0.83
**Baseline self-rated health**				**0.001**
Poor/Average, %	26.4	19.4	23.4	
Good, %	44.2	40.3	38.9	
Very good/Excellent %	28.8	40.3	37.7	
**Baseline energy from grocery stores**	1,921±48	1,448±30	1,254±30	**<0.001**
**Follow-up energy from grocery stores**	1,424±35	1,494±30	1,779±40	**<0.001**
Δ **% energy from grocery stores** (X ± SE)	+0.53±0.34	0.08±0.27	-0.39±0.30	0.11

*Abbreviations*: Δ = Annual rate of change; HANDLS = Healthy Aging in Neighborhood of Diversity across the Lifespan; HS = High School; MVD = Monetary value of the diet; PIR = Poverty Income Ratio; SE = Standard Error; T = tertile; WRAT-3 = Wide Range Achievement Test, 3^rd^ revision.

^1^ The monetary value of the diet (MVD) was estimated for each HANDLS wave using the HOMESCAN database at the annual and quarterly level for each food group. This was summed across individual dietary recall and averaged across individual participant in each wave. The annual rate of change in MVD (ΔMVD) is the difference between follow-up and baseline MVD divided by the time elapsed between baseline and follow-up. A similar calculation was done for Δ Energy intake (kcal/d/y) and Δ % energy from grocery stores.

^2^ P-value from one-way ANOVA (continuous variables) or from χ^2^ test (categorical variables). PIR = Poverty Income Ratio; SE = Standard Error; WRAT-3 = Wide Range Achievement Test, version 3.

^3^Researchers own analyses and calculations based in part on data reported by Nielsen through its Homescan Service for the food and beverage categories for the years 2004–2013, for the US market Nielsen data is licensed from The Nielsen Company, 2016 The conclusions drawn from the Nielsen data are those of the Researchers and do not reflect the views of Nielsen. Nielsen is not responsible for and was not involved in analyzing and preparing the results reported herein.

**S1 Table** presents means of baseline, follow-up and longitudinal annual rates of change in diet quality by sex, race and poverty status. Both baseline and follow-up total scores on HEI-2010 indicated better quality among women compared to men, while the reverse was true for MAR. For both diet quality indices, individuals above poverty scored higher than participants living below poverty. An additional differential was observed for MAR, whereby Whites had a better diet quality compared with African-Americans. However, when examining annual rates of change in dietary quality, ΔHEI-2010 was comparable across socio-demographic groups, while ΔMAR was higher among women and individuals above poverty. Specifically, MAR was found to be increasing at a faster pace among women compared to men in terms of intakes of vitamin A, vitamin C, niacin, iron, though a slower pace was noted for zinc NAR. In terms of poverty status differentials, MAR was found to increase at a faster pace among adults above poverty compared to below poverty, mainly through a higher ΔNAR in vitamin A, vitamin E, vitamin B-12, riboflavin, folate, calcium and magnesium. Other socio-demographic differentials, including differences in specific ΔHEI-2010 components by race were inconsistent.

**Tables 2** and **3** show adjusted associations between annual rate of change in MVD tertiles and annual rates of change in the key diet quality indices, stratifying by sex, race and poverty status. ΔMVD was positively associated with both ΔHEI-2010 and ΔMAR, and with a consistently stronger association among individuals above poverty, specifically for the total proteins and empty calories components of HEI-2010 and several NARs (vitamins C, E, B-6 and Zinc). ΔMVD-ΔMAR association was stronger in women, mainly influenced by ΔMVD’s positive associations with B-vitamins, copper, calcium, magnesium and phosphorus NARs. ΔMVD-Δvitamin D NAR’s positive relationship was stronger among Whites, while ΔMVD-Δvitamin B-12 NAR’s association was stronger among African-Americans. Key associations between ΔMVD tertiles and changes in the two diet quality indices are illustrated in **S2 through S4 Figs** using predictive margins from the mixed-effects regression models, stratifying by sex, race and poverty status.

**Table 2:** Annual rate of change in MVD tertiles as predictors of annual rate of change in the HEI-2010 (total score and components), stratifying by sex, race and poverty status: Multiple ordinary least square regression models, HANDLS 2004−2013^5^.

	ΔMVD tertiles ($/d/y) ^1^		
	β±SE ^1^ (T_2_ vs. T_1_)	β±SE ^1^ (T_3_ vs. T_1_)	P-trend ^2^
Δ **HEI-2010 total score**			
Overall	+0.71±0.19***	+1.33±0.22***	**<0.001**
Men	+0.56±0.28*	+1.29±0.31***	**<0.001**
Women	+0.85±0.27**	+1.44±0.32***	**<0.001**
Whites	+0.87±0.33**	+1.70±0.37***	**<0.001**
AA	+0.61±0.23**	+1.08±0.28***	**<0.001**
Above poverty	+0.84±0.26**	+1.76±0.30***^,^^4^	**<0.001**^4^
Below poverty	+0.47±0.27†	+0.56±0.34†	0.097
Δ **Total vegetables**			
Overall	+0.02±0.03	+0.06±0.03†	0.061
Men	+0.04±0.05	+0.07±0.05	0.14
Women	+0.02±0.04	+0.06±0.04	0.15
Whites	-0.01±0.04	+0.05±0.05	0.35
AA	+0.04±0.03	+0.07±0.04†	0.072
Above poverty	+0.01±0.04	+0.08±0.04†	0.049
Below poverty	+0.02±0.04	+0.01±0.05	0.79
Δ **Greens and beans**			
Overall	+0.02±0.03	+0.09±0.04**	**0.008**
Men	-0.03±0.04	+0.00±0.05^3^	0.97 ^3^
Women	+0.07±0.04	+0.17±0.05**	**0.001**
Whites	-0.07±0.05	+0.03±0.06	0.57
AA	+0.09±0.04*	+0.15±0.05**	**0.001**
Above poverty	-0.02±0.04	+0.09±0.05	0.050
Below poverty	+0.06±0.04	+0.09±0.05†	0.088
Δ **Total fruit**			
Overall	+0.06±0.03†	+0.09±0.03*	**0.013**
Men	+0.06±0.04	+0.10±0.05*	**0.041**
Women	+0.06±0.04	+0.09±0.05†	0.091
Whites	+0.10±0.05*	+0.14±0.05**	**0.009**
AA	+0.02±0.04	+0.02±0.05	0.64
Above poverty	+0.11±0.04**	+0.16±0.05**	**0.001**
Below poverty	-0.01±0.04	-0.03±0.05	0.58
Δ **Whole fruit**			
Overall	+0.09±0.03**	+0.22±0.04***	**<0.001**
Men	+0.11±0.04*	+0.23±0.05***	**<0.001**
Women	+0.08±0.04†	+0.22±0.05***	**<0.001**
Whites	+0.14±0.05**	+0.26±0.06***	**<0.001**
AA	+0.04±0.04	+0.17±0.04***	**<0.001**
Above poverty	+0.13±0.04**	+0.27±0.05***	**<0.001**
Below poverty	+0.02±0.04	+0.14±0.05**	**0.005**
Δ **Whole grains**			
Overall	+0.10±0.05*	+0.14±0.05*	**0.015**
Men	+0.15±0.08*	+0.06±0.08	0.45
Women	+0.07±0.07	0.20±0.09*	**0.018**
Whites	+0.18±0.09†	+0.20±0.10*	**0.051**
AA	+0.05±0.06	+0.09±0.07	0.19
Above poverty	+0.15±0.07*	+0.17±0.08*	**0.047**
Below poverty	+0.05±0.07	+0.12±0.08	0.15
Δ **Dairy**			
Overall	+0.22±0.05***	+0.44±0.06***	**<0.001**
Men	+0.25±0.08**	+0.48±0.09***	**<0.001**
Women	+0.21±0.07**	+0.42±0.08***	**<0.001**
Whites	+0.24±0.09*	+0.54±0.11***	**<0.001**
AA	+0.19±0.06**	+0.34±0.07***	**<0.001**
Above poverty	+0.17±0.07*	+0.41±0.08***	**<0.001**
Below poverty	+0.31±0.07***	+0.48±0.09***	**<0.001**
Δ **Total protein foods**			
Overall	+0.04±0.02†	+0.04±0.02†	0.087
Men	+0.05±0.03†	+0.07±0.03*	**0.037**
Women	+0.01±0.03	+0.00±0.03	0.91
Whites	+0.04±0.04	+0.06±0.04	0.14
AA	+0.03±0.02	+0.03±0.03	0.24
Above poverty	+0.07±0.03*	+0.09±0.03**^,^^4^	**0.005**^4^
Below poverty	-0.01±0.03	-0.04±0.04	0.28
Δ **Seafood and plant proteins**			
Overall	+0.09±0.03**	+0.05±0.04	0.24
Men	+0.05±0.05	+0.03±0.06	0.59
Women	+0.11±0.04*	+0.06±0.05	0.33
Whites	+0.11±0.06†	+0.05±0.06	0.42
AA	+0.09±0.04*	+0.05±0.05	0.31
Above poverty	+0.09±0.05†	+0.04±0.05	0.44
Below poverty	+0.08±0.05†	+0.06±0.06	0.30
Δ **Fatty acids**			
Overall	-0.11±0.06†	-0.05±0.07	0.50
Men	-0.15±0.09	-0.09±0.10	0.38
Women	-0.08±0.08	-0.02±0.09	0.85
Whites	-0.09±0.11	+0.10±0.11	0.38
AA	-0.12±0.07†	-0.15±0.08†	0.08
Above poverty	-0.07±0.08	+0.02±0.09	0.84
Below poverty	-0.18±0.08*	-0.15±0.10	0.12
Δ **Sodium**			
Overall	-0.08±0.06	-0.21±0.07**	**0.002**
Men	-0.13±0.09	-0.12±0.10	0.22
Women	-0.04±0.08	-0.27±0.10**	**0.005**
Whites	-0.03±0.10	-0.35±0.11**	**0.002**
AA	-0.09±0.07	-0.10±0.09	0.27
Above poverty	-0.15±0.08†	-0.31±0.09**	**0.001**
Below poverty	+0.01±0.09	-0.07±0.11	052
Δ **Refined grains**			
Overall	+0.18±0.06**	+0.30±0.07***	**<0.001**
Men	+0.12±0.09	+0.13±0.10	0.20
Women	+0.28±0.08***	+0.53±0.09***	**<0.001**
Whites	+0.17±0.10	+0.27±0.12*	**0.021**
AA	+0.23±0.07***	+0.41±0.08***	**<0.001**
Above poverty	+0.18±0.08*	+0.32±0.09***	**<0.001**
Below poverty	+0.17±0.08*	+0.27±0.10**	**0.007**
Δ **Empty calories**			
Overall	+0.07±0.09	+0.16±0.15	0.15
Men	+0.03±0.15	+0.32±0.17†	0.064
Women	+0.05±0.12	-0.02±0.15	0.86
Whites	+0.08±0.16	+0.35±0.18†	0.055
AA	+0.05±0.11	-0.02±0.13	0.87
Above poverty	+0.15±0.12	+0.43±0.14**^,^^4^	**0.002**^,^^4^
Below poverty	-0.07±0.14	-0.33±0.17*	**0.049**

*Abbreviations*: Δ = Annual rate of change; HEI-2010 = Healthy Eating Index, 2010 version; HANDLS = Healthy Aging in Neighborhood of Diversity across the Lifespan; MVD = Monetary value of the diet; SE = Standard Error.

***P<0.001

**P<0.010

*P<0.05

†P<0.10 for null hypothesis that β = 0 (i.e. T_2_ vs. T_1_ and/or T_3_ vs. T_1_).

^1^ Values are regression coefficients and their standard errors (β±SE) from a multivariable linear regression model with Y = annual rate of change in 2010-HEI (or components) or MAR (or components) and the key predictor being tertile of annual rate of change in MVD, contrasting the middle tertile with the lowest tertile (T_2_ vs. T_1_) and the uppermost tertile with the lowest tertile (T_3_ vs. T_1_). Models were adjusted for baseline age, sex, race, poverty status, educational attainment, literacy, employment status, current smoking status, current drug use, body mass index, self-rated health, annual rates of change in total energy intake and in % energy from grocery stores.

^2^ P-trend was derived from a similar model as in a, but with the key predictor MVD tertiles entered as a single ordinal variable rather than two dummy variables.

^3^ P<0.05 for null hypothesis that the term sex*MVD = 0 in a separate un-stratified regression model in which this interaction term was added.

^4^ P<0.05 for null hypothesis that the term pov*MVD = 0 in a separate un-stratified regression model in which this interaction term was added.

^5^Researchers own analyses and calculations based in part on data reported by Nielsen through its Homescan Service for the food and beverage categories for the years 2004–2013, for the US market Nielsen data is licensed from The Nielsen Company, 2016 The conclusions drawn from the Nielsen data are those of the Researchers and do not reflect the views of Nielsen. Nielsen is not responsible for and was not involved in analyzing and preparing the results reported herein.

**Table 3:** Annual rate of change in MVD tertiles as predictors of annual rate of change in MAR and NAR, stratifying by sex, race and poverty status: Multiple ordinary least square regression models, HANDLS 2004−2013^5^.

	ΔMVD tertiles ($/d/y) ^1^		
	β±SE ^1^ (T_2_ vs. T_1_)	β±SE ^1^ (T_3_ vs. T_1_)	P-trend ^2^
Δ **MAR score**			
Overall	+1.40±0.19***	+2.90±0.20***	**<0.001**
Men	+1.08±0.26***^,^ ^3^	+2.20±0.30***^,^ ^3^	**<0.001** ^3^
Women	+1.45±0.26***	+2.93±0.32***	**<0.001**
Whites	+1.31±0.32***	+3.17±0.35***	**<0.001**
AA	+1.39±0.23***	+2.60±0.28***	**<0.001**
Above poverty	+1.36±0.26***	+3.29±0.29***^,^^5^	**<0.001**^,^^5^
Below poverty	+1.44±0.27***	+2.22±0.34***	**<0.001**
Δ **Vitamin A, NAR**			
Overall	+1.89±0.53***	+4.61±0.62***	**<0.001**
Men	+1.89±0.84*	+3.46±0.94***	**<0.001**
Women	+2.01±0.71**	+5.63±0.86***	**<0.001**
Whites	+0.16±0.91^4^	+3.60±1.02***	**<0.001**
AA	+3.09±0.65***	+5.39±0.80***	**<0.001**
Above poverty	+1.40±0.75†	+4.76±0.84***	**<0.001**
Below poverty	+2.61±0.75**	+4.32±0.93***	**<0.001**
Δ **Vitamin C, NAR**			
Overall	+0.91±0.67	+1.01±0.78	0.20
Men	+0.24±1.04	-0.44±1.16	0.72
Women	+1.87±0.90*	+2.87±1.10**	**0.010**
Whites	+1.00±1.09	+2.57±1.22*	**0.034**
AA	+0.87±0.86	-0.03±1.04	0.98
Above poverty	+1.73±0.93†	+2.69±1.04*^,^^5^	**0.010**^,^^5^
Below poverty	-0.30±0.97	-1.74±1.21	0.15
Δ **Vitamin D, NAR**			
Overall	+2.84±0.44***	+4.09±0.51***	**<0.001**
Men	+1.63±0.68*	+3.04±0.76***	**<0.001**
Women	+3.94±0.58***	+5.23±0.71***	**<0.001**
Whites	+2.65±0.59***	+4.85±0.65***^,^ ^4^	**<0.001**^4^
AA	+3.09±0.62***	+3.90±0.75***	**<0.001**
Above poverty	+2.15±0.54***	+3.48±0.60***	**<0.001**
Below poverty	+3.86±0.74***	+5.08±0.91***	**<0.001**
Δ **Vitamin E, NAR**			
Overall	+0.14±0.38	+1.32±0.44**	**0.003**
Men	-0.33±0.60	+1.20±0.67†	0.083
Women	+0.23±0.50	+0.92±0.61	0.13
Whites	+0.50±0.67	+2.60±0.75**	**0.001**
AA	-0.18±0.44	+0.30±0.54	0.58
Above poverty	+0.82±0.54	+2.64±0.61***^,^^5^	**<0.001**^,^^5^
Below poverty	-0.96±0.50†	-0.93±0.61	0.13
Δ **Vitamin B-6, NAR**			
Overall	+2.03±0.35***	+3.80±0.41***	**<0.001**
Men	+1.56±0.47**^, 3^	+3.40±0.52***^,^^3^	**<0.001** ^3^
Women	+2.11±0.51***	+3.40±0.62***	**<0.001**
Whites	+1.99±0.60**	+4.11±0.67***	**<0.001**
AA	+1.97±0.43***	+3.50±0.53***	**<0.001**
Above poverty	+2.11±0.47***	+4.69±0.52***^,^^5^	**<0.001**^5^
Below poverty	+1.82±0.55**	+2.19±0.68**	**0.001**
Δ **Vitamin B-12, NAR**			
Overall	+1.13±0.35**	+2.74±0.41***	**<0.001**
Men	+0.52±0.48^, 3^	+1.89±0.53***^,^^3^	**<0.001** ^3^
Women	+1.35±0.50**	+3.01±0.61***	**<0.001**
Whites	+0.58±0.53	+2.55±0.59***	**<0.001** ^4^
AA	+1.42±0.47**	+2.79±0.57***	**<0.001**
Above poverty	+1.00±0.49*	+3.16±0.54***	**<0.001**
Below poverty	+1.30±0.51*	+2.04±0.63**	**0.001**
Δ **Thiamin, NAR**			
Overall	+1.16±0.32***	+2.47±0.37***	**<0.001**
Men	+1.29±0.46**	+2.02±0.52***^,^ ^3^	**<0.001** ^3^
Women	+0.67±0.44	+1.91±0.54***	**<0.001**
Whites	+1.33±0.51*	+2.52±0.57***	**<0.001**
AA	+0.91±0.42*	+2.15±0.51***	**<0.001**
Above poverty	+1.05±0.43	+2.58±0.48***	**<0.001**
Below poverty	+1.65±0.50**	+2.30±0.62***	**<0.001**
Δ **Riboflavin, NAR**			
Overall	+0.75±0.24**	+2.05±0.28***	**<0.001**
Men	+0.32±0.36	+1.41±0.41**^,^ ^3^	**0.001** ^3^
Women	+0.84±0.33*	+2.07±0.40***	**<0.001**
Whites	+0.51±0.34^4^	+1.61±0.38***^,^ ^4^	**<0.001**
AA	+0.80±0.34*	+2.05±0.41***	**<0.001**
Above poverty	+0.65±0.32*	+2.26±0.36***	**<0.001**
Below poverty	+1.00±0.38**	+1.76±0.47***	**<0.001**
Δ **Niacin, NAR**			
Overall	+1.33±0.28***	+2.22±0.32***	**<0.001**
Men	+0.71±0.37†^, 3^	+1.72±0.42***^,^ ^3^	**<0.001** ^3^
Women	+1.35±0.38***	+1.76±0.47***	**<0.001**
Whites	+1.33±0.48**	+2.56±0.54***	**<0.001**
AA	+1.27±0.33***	+1.88±0.41***	**<0.001**
Above poverty	+1.00±0.38**	+2.40±0.42***	**<0.001**
Below poverty	+1.66±0.40***	+1.80±0.50***	**<0.001**
Δ **Folate, NAR**			
Overall	+1.52±0.38***	+3.47±0.44***	**<0.001**
Men	+1.73±0.52**	+2.95±0.59***^,^ ^3^	**<0.001** ^3^
Women	+1.03±0.54†	+2.90±0.65***	**<0.001**
Whites	+1.64±0.67*	+3.88±0.74***	**<0.001**
AA	+1.20±0.45**	+2.69±0.55***	**<0.001**
Above poverty	+1.18±0.53*	+3.74±0.59***	**<0.001**
Below poverty	+1.98±0.54***	+2.99±0.67***	**<0.001**
Δ **Iron, NAR**			
Overall	+1.08±0.34**	+2.74±0.40***	**<0.001**
Men	+0.19±0.27^, 3^	+0.52±0.31†^,^ ^3^	**<0.001**
Women	+1.50±0.50**	+3.04±0.61***	**<0.001**
Whites	+1.12±0.59†	+2.66±0.66***	**<0.001**
AA	+0.96±0.41*	+2.65±0.50***	**<0.001**
Above poverty	+0.93±0.47*	+2.72±0.53***	**<0.001**
Below poverty	+1.27±0.49*	+2.60±0.61***	
Δ **Copper, NAR**			
Overall	+1.23±0.28***	+2.86±0.33***	**<0.001**
Men	+1.03±0.39**	+2.10±0.43***	**<0.001** ^3^
Women	+1.04±0.39**	+2.63±0.48***	**<0.001**
Whites	+0.99±0.47*	+2.83±0.52***	**<0.001**
AA	+1.30±0.35***	+2.80±0.42***	**<0.001**
Above poverty	+1.57±0.39***	+3.26±0.43***	**<0.001**
Below poverty	+0.73±0.40†	+2.22±0.50***	**<0.001**
Δ **Zinc, NAR**			
Overall	+1.75±0.39***	+3.94±0.45***	**<0.001**
Men	+2.44±0.60***	+5.11±0.68***	**<0.001**
Women	+0.76±0.50	+2.06±0.61**	**0.001**
Whites	+2.29±0.64***	+4.78±0.72***	**<0.001**
AA	+1.14±0.47*	+3.09±0.58***	**<0.001**
Above poverty	+2.66±0.53***^,^^5^	4.94±0.60***^,^^5^	**<0.001**^,^^5^
Below poverty	+0.60±0.55	+2.37±0.68**	**0.001**
Δ **Calcium, NAR**			
Overall	+1.98±0.41***	+4.08±0.47***	**<0.001**
Men	+2.02±0.61**	+3.75±0.68***^,^ ^3^	**<0.001** ^3^
Women	+1.62±0.56**	+3.76±0.68***	**<0.001**
Whites	+2.40±0.65***	+4.72±0.73***	**<0.001**
AA	+1.64±0.52**	+3.39±0.64***	**<0.001**
Above poverty	+1.13±0.55*^,^^5^	+4.04±0.61***	**<0.001**
Below poverty	+3.11±0.61***	+4.06±0.75***	**<0.001**
Δ **Magnesium, NAR**			
Overall	+2.10±0.27***	+3.53±0.32***	**<0.001**
Men	+1.70±0.40***	+2.67±0.45***^,^ ^3^	**<0.001** ^3^
Women	+2.32±0.38***	+4.15±0.46***	**<0.001**
Whites	+2.19±0.48***	+3.30±0.53***	**<0.001**
AA	+2.02±0.33***	+3.73±0.40***	**<0.001**
Above poverty	+1.80±0.37***	+3.65±0.42***	**<0.001**
Below poverty	+2.52±0.40***	+3.36±0.50***	**<0.001**
Δ **Phosphorus, NAR**			
Overall	+0.59±0.21**	+1.47±0.24***	**<0.001**
Men	+0.30±0.24^3^	+0.56±0.27*^,^ ^3^	**0.040** ^3^
Women	+0.55±0.31†	+1.55±0.38***	**<0.001**
Whites	+0.23±0.33	+1.50±0.36***	**<0.001**
AA	+0.75±0.27**	+1.29±0.33***	**<0.001**
Above poverty	+0.59±0.29*	+1.66±0.32***	**<0.001**
Below poverty	+0.55±0.31†	+1.07±0.38**	**0.005**

*Abbreviations*: Δ = Annual rate of change; HANDLS = Healthy Aging in Neighborhood of Diversity across the Lifespan; MAR = Mean Adequacy Ratio; MVD = Monetary value of the diet, NAR = Nutrient Adequacy Ratio; SE = Standard Error.

***P<0.001

**P<0.010

*P<0.05

†P<0.10 for null hypothesis that β = 0 (i.e. T_2_ vs. T_1_ and/or T_3_ vs. T_1_).

^1^ Values are regression coefficients and their standard errors (β±SE) from a multivariable linear regression model with Y = annual rate of change in 2010-HEI (or components) or MAR (or components) and the key predictor being tertile of annual rate of change in MVD, contrasting the middle tertile with the lowest tertile (T_2_ vs. T_1_) and the uppermost tertile with the lowest tertile (T_3_ vs. T_1_). Models were adjusted for baseline age, sex, race, poverty status, educational attainment, literacy, employment status, current smoking status, current drug use, body mass index, self-rated health, annual rates of change in total energy intake and in % energy from grocery stores.

^2^ P-trend was derived from a similar model as in a, but with the key predictor MVD tertiles entered as a single ordinal variable rather than two dummy variables.

^3^ P<0.05 for null hypothesis that the term sex*MVD = 0 in a separate un-stratified regression model in which this interaction term was added.

^4^ P<0.05 for null hypothesis that the term race*MVD = 0 in a separate un-stratified regression model in which this interaction term was added.

^5^ P<0.05 for null hypothesis that the term pov*MVD = 0 in a separate un-stratified regression model in which this interaction term was added.

^6^Researchers own analyses and calculations based in part on data reported by Nielsen through its Homescan Service for the food and beverage categories for the years 2004–2013, for the US market Nielsen data is licensed from The Nielsen Company, 2016 The conclusions drawn from the Nielsen data are those of the Researchers and do not reflect the views of Nielsen. Nielsen is not responsible for and was not involved in analyzing and preparing the results reported herein.

## Discussion

This research is the first to link annual rates of change (Δ) in MVD to its dietary quality counterpart using the HEI-2010 and MAR scores simultaneously among urban US adults, while systematically examining differential associations across sex, race and poverty status. Among key findings, ΔHEI-2010 was comparable across socio-demographic groups, while ΔMAR was higher among women and individuals above poverty. Adjusting for key covariates, ΔMVD was positively associated in both ΔHEI-2010 and ΔMAR, and with a consistently stronger association among individuals above poverty, specifically for the total proteins and empty calories components of HEI-2010 and several nutrient adequacy ratios (NARs: vitamins C, E, B-6 and Zinc). ΔMVD-ΔMAR association was stronger in women, mainly influenced by ΔMVD’s positive associations with B-vitamins, copper, calcium, magnesium and phosphorus NARs. ΔMVD-Δvitamin D NAR’s positive relationship was stronger among Whites, while ΔMVD-Δvitamin B-12 NAR’s association was stronger among African-Americans.

Although this is the first study examining this research question using a longitudinal design in a US population, another very recent longitudinal study conducted among Spanish adults found that increasing the average daily dietary cost from 3·68(SD0.0·89)€/8·36 MJ to 4·97(SD1·16)€/8·36 MJ during the study period was associated with significant improvements in diet quality, as reflected by the Mediterranean Diet Score (MDS), (Δ ED and Δ MDS-rec; P<0·0001). A 1€ increase in monetary diet cost per 8·36 MJ was linked to a decrease of an estimated 0·3 kg in body weight (P = 0·02) and a 0·1 kg/m2 in BMI (P = 0·04). An attenuation in those associations was observed after adjustment for changes in diet quality indicators.[59]

Another observational panel data study of UK adults concluded that a lower expenditure on food is likely a key contributor to less-healthy food choices among lower socioeconomic groups, through a series of mediation analyses. Specifically, 63% of the total socioeconomic differences in choices of less-healthy foods/beverages were explained through a pathway involving expenditure, with a similar mediation proportion estimated at 36% for fruit and vegetables. Those figures were attenuated to 53% and 31%, respectively after adjustment for supermarket choice.[58] In addition, two intervention studies mirrored those findings among French adults.[56,57] For instance, simulating subsidies among French adult women triggered improvements in nutritional quality (fruit and vegetable quantities increased, ED decreased, the MAR increased), though the improvement magnitudes were often reduced among lower income women.[57]

Previous cross-sectional studies have corroborated our findings. For instance, a large study of women enrolled in the US Nurses’ Health Study (N = 78,191) found that energy-adjusted MVD (uppermost vs. lowest quintile) was associated with a 30 point higher Alternative Healthy Eating Index score.[37] In another large cross-sectional study that restricted its sample to adult UK women (N = 35,000), dietary diversity and healthful dietary patterns were linked to a higher MVD, while SES among women had a positive association with the likelihood of a healthful dietary pattern. (44) Furthermore, another study found MVD to be a key mediator in the pathway linking income to diet quality, and education a moderator in that pathway. [42] Using the same sample (N = 1,266), another study reported MVD to be directly associated with dietary intakes of fiber, several vitamins (A, C, D, E, and B-12, β-carotene, folate), and selected minerals (iron, calcium, potassium and magnesium); contrasted with an inverse relationship between MVD and intakes of saturated fats, trans fats, and added sugars.[46] Among French adults, belonging to the highest MAR tertile was associated with the lowest dietary energy density and the highest diet costs,[30] a finding that was replicated by another cross-sectional study.[42]

Two studies by Rehm and colleagues using NHANES 2001–2002 and 2007–2010 corroborated findings from other large cross-sectional studies regarding the direct association between MVD and dietary quality, specifically as measured by the HEI-2005 and HEI-2010 indices. In their first study (NHANES 2001–2002), they reported that higher MVD was strongly associated with consuming more servings of fruit and vegetables and fewer calories derived from solid fat, alcoholic beverages and added sugars, as well as the total HEI-2005 score.[44] Using recent waves of NHANES (2007–2010) and the HEI-2010, Rehm and colleagues found that lower dietary costs were associated with lower consumption of vegetables, fruits, whole grains, and seafood, and higher consumption of refined grains, solid fat, alcohol and added sugars. The direct relationship between MVD and the total score on HEI-2010 was stronger among women, a finding that is similar to ours. Individual studies, including our previous cross-sectional analysis of HANDLS, also indicated that MVD’s positive association with dietary quality associations was stronger among US women compared to men. [33,34,44,54]

Moreover, in our study, the annual rate of change in the sodium moderation component of the HEI-2010 was inversely associated with ΔMVD, after adjustment for energy intake and other key confounders. This finding was comparable to our previous cross-sectional analysis of HANDLS,[10] as well as another study which reported a positive association between MVD and biomarker-based estimates of protein, potassium and sodium intake in young Japanese women. In the latter study, the monetary costs of vegetables and fish were main mediators.[35]

Pooling results from 24 studies and from 10 countries, a recent meta-analysis suggested that there were marked pricing differences between food groups, which ultimately increased the cost of healthful diets. Nevertheless, price differences had a weaker effect on nutrient-based patterns.[60] In addition, a more recent review came to a similar conclusion, whereby acceptable healthier diets were uniformly associated with higher costs, with food budgets in poverty being insufficient to ensure optimum diets.[55]

Among many of our study’s strengths, it is the first to utilize the HEI-2010 and MAR scores to test MVD’s association with overall dietary quality, while using longitudinal measurements of dietary intakes and their estimated MVD. Specifically, annual rates of change in those diet quality indices were linked to the annual rate of change in their estimated cost, using time-varying food price indices at the quarterly levels for each year that were assigned and linked to several food groups. Furthermore, it is among the few studies to examine differential associations between diet quality and MVD across race, sex and income groups. Our findings can be extrapolated to several African American and White populations, as HANDLS is representative of 14 urban settings across the United States. An improvement over our previous cross-sectional analysis of the HANDLS study,[10] is our assignment of food prices that were specific to Baltimore City, were measured to the nearest quarter and year, and that were deflated to a specific time point (the first quarter of 2004).

Nevertheless, our study findings should be interpreted with caution, given several important limitations. First, using food prices to estimate MVD may mildly underestimate actual food expenditures. [15] Second, there is evidence that the poor, given their lower volume purchases and reduced ability to travel, had a higher MVD.[82] This suggests that our key finding of the income differential in the association between ΔMVD and change in diet quality is potentially underestimated.[83,84] In terms of MVD estimation, some FNDDS food codes used to obtain food price indices lacked specificity needed to provide an exact match to foods as purchased; for example, a “burrito with chicken” may be reported for burritos purchased frozen, refrigerated, or prepared from a meal kit. Another limitation is that foods without barcodes, such as unpackaged fresh produce or meat sold by weight, are excluded from the database; prices for corresponding food groups are generated only from packaged items, over-representing frozen and canned items while under-representing fresh products. Homescan participants do not report foods purchased from fast food eateries, restaurants, and other away-from-home sources, and prices of foods purchased from stores may differ from prices these away-from-home sources. In addition, as stated earlier, imputations were conducted in order to estimate food price indices for certain FNDDS codes that did not fit into the pre-assigned 42 food groups.

In conclusion, we found a positive overall association between rate of change in MVD and that of indices of dietary quality. For both HEI-2010 and MAR, participants above poverty had a stronger longitudinal association between MVD and overall dietary quality as opposed to participants below poverty. Our findings also indicated that diet quality based on the HEI-2010 is far from being compliant with the Dietary Guidelines at both waves, whereby compliance is defined by a cut-point of 80 or higher. While a higher income can be a driving force for the effectiveness of MVD in reaching this level of compliance, nutrition education is another force that would influence people’s decision-making regarding food venues and dietary choices. Since this study is observational, future intervention studies are warranted in urban populations to examine the differential effects of MVD on dietary quality across sex, race and poverty status.

## Supporting information

S1 Method HomeScan data description. (DOCX)

S2 Method Food group description. (DOCX)

S1 Table (DOCX)

S1 Fig (PPTX)

S2 Fig (PPTX)

S3 Fig (PPTX)

S4 Fig (PPTX)

## Abbreviations

Δ: Annual rate of change
DQ: Diet Quality
HEI-2010: Healthy Eating Index, 2010 version
FNDDS: Food and Nutrient Database for Dietary Studies
HANDLS: Healthy Aging in Neighborhood of Diversity across the Life Span
HS: High School
MAR: Mean Adequacy Ratio, MVD = Monetary value of the diet, NAR = Nutrient Adequacy Ratio, NHANES = National Health and Nutrition Examination Surveys
PIR: Poverty Income Ratio
RDA: Recommended Dietary Allowance
SE: Standard Error
USDA: US Department of Agriculture
WRAT-3: Wide Range Achievement Test, 3^rd^ revision
