# Supplementary material for: Longitudinal change in the diet's monetary value is associated with its change in quality and micronutrient adequacy among urban adults
Source: PLoS One. 2018 Oct 12;13(10):e0204141. doi: 10.1371/journal.pone.0204141 (PMC6193582; doi:10.1371/journal.pone.0204141)
Supplement: S1 Method — (DOCX) [file pone.0204141.s001.docx]

**Supplemental methods 1: HomeScan data description**

The Homescan panel is a nationwide sample of US households that record all packaged foods and beverages purchased from grocery stores, supermarkets, and other retail food stores continuously throughout the year. Households are followed prospectively and must report purchases for at least 10 months per year. The sample includes approximately 40,000-60,000 US households each year from 76 geographic markets, and Nielsen provides projection factor weights to generate nationally representative estimates.([1](#_ENREF_1)) Household members scan the Universal Product Code barcode on each purchased item after each shopping trip using a handheld scanner and report the quantity purchased. Methods for reporting price paid depend on the store where the purchase takes place. For most products, Nielsen imputes the price paid from store-level point-of-sales data (“ScanTrack”) as the average price paid for the product from that store for the given week and market.([2](#_ENREF_2)) However, for items purchased from stores not covered by ScanTrack, households must manually record the price paid; if the reported price is outside of the typical range, Nielsen replaces the reported value with the median regional price.([2](#_ENREF_2))

**References**:

1. Muth M, Siegel P, Zhen C. ERS Data Quality Study Design. Research Triangle Park, NC: RTI International. USDA, Economic Research Service, 2007.

2. Einav L, Leibtag E, Nevo A. On the accuracy of Nielsen Homescan data. Washington, DC: USDA, Economic Research Service, 2008.
