# Supplementary material for: Longitudinal change in the diet's monetary value is associated with its change in quality and micronutrient adequacy among urban adults
Source: PLoS One. 2018 Oct 12;13(10):e0204141. doi: 10.1371/journal.pone.0204141 (PMC6193582; doi:10.1371/journal.pone.0204141)
Supplement: S2 Method — (DOCX) [file pone.0204141.s002.docx]

**Supplemental methods 2: Food group description**

| **FG UNC** | **UNC Description of FG** | **HANDLS code for closest FG** |
| --- | --- | --- |
| 1 | cheese | 24,25,26 |
| 2 | yogurt |  |
| 3 | dairy products, other |  |
| 4 | dairy-based desserts | 30,31 |
| 5 | meat | 32,36,38,39 |
| 6 | meat, breaded | 33,35,37 |
| 7 | processed meat | 40 |
| 8 | Eggs | 44 |
| 9 | Legumes | 45 |
| 10 | Nuts and nut products | 57 |
| 11 | Bread and bread products | 1,2 |
| 12 | TORTILLAS, TACO SHELLS, AND WRAPS |  |
| 13 | QUICK BREADS |  |
| 14 | GRAIN-BASED DESSERTS |  |
| 15 | Grain-based bars |  |
| 16 | Pasta and rice | 4,5,6 |
| 17 | Cereal | 7 |
| 18 | Fruit | 13,14,15,16 |
| 19 | Fruit dish | 17 |
| 20 | Vegetables | 18,19,21 |
| 21 | Starchy vegetables | 20 |
| 22 | Fried potatoes |  |
| 23 | Fats and oils | 54,55,56 |
| 24 | Sweeteners | 52 |
| 25 | CANDY AND SWEET SNACKS | 51,53 |
| 26 | Baking products | 11 |
| 27 | Salt and seasoning | 60 |
| 28 | Soups and stews | 14,41 |
| 29 | Salty snacks | 8,9 |
| 30 | SAUCES, DIPS, AND CONDIMENTS |  |
| 31 | Baby food | 12 |
| 32 | MIXED DISH, REFRIGERATED |  |
| 33 | MIXED DISH, FROZEN | 46 |
| 34 | MIXED DISH, CANNED/SHELF-STABLE |  |
| 35 | MIXED DISH, INSTANT/MIX |  |
| 36 | Water | 61 or closest neighbor |
| 37 | Coffee and tea | 47 |
| 38 | SSB | 48,49 |
| 39 | FRUIT AND VEGETABLE JUICE |  |
| 40 | Milk | 22,23 |
| 41 | COCOA AND SWEETENED MILK BEVERAGES |  |
| 42 | MILK SUBSTITUTES AND MILK BEVERAGES | 58 |

|  | Use closest neighbor imputation instead if imputed |
| --- | --- |
|  | Use HANDLS code if imputed; UNC code otherwise |

**HANDLS 61 food groups.**

***GRAINS***

1. (1) Refined breads, (3) Multigrain breads, (5) Low sodium breads, (6) Refined Quick breads
2. (2) 100% whole wheat breads, (4) High fiber, reduced calorie breads, (7) Whole wheat quick breads, (10) Whole wheat pasta without added fat, (11) Whole wheat pasta with added fat, (12) Brown rice without fat added, (13) Brown rice with fat added
3. (18) Whole grain cooked cereals no fat added, (19) Whole grain cooked cereals with fat added, (25) Whole wheat crackers, (21) High fiber cereals
4. (8) Pastas without added fat, (9) Pastas with added fat, (154) Pasta with meat, (155) Pasta without meat
5. (14) Cereals and white rice without added fat, (15) Cereals and white rice with added fat, (156) Rice dishes with meat, (157) Rice dishes without meat
6. (16) Cooked cereals with no fat, (17) Cooked cereals with fat added

7. (20) Ready to eat cereals, (37) Breakfast bars

8. (22) Regular crackers, (26) Salty snacks (chips, pretzels, popcorn, chips)

9 (23) Reduced fat crackers, (27) Low sodium snacks (pretzels, crackers, chips, popcorn), (28) Reduced fat potato chips

10. (24) Sweet crackers (graham, animal), (29) Cakes, (31) Doughnuts, (32) Cookies, (34) Pies (excludes fruit pies), (36) Pastries

11. (30) Diet cakes and pastries, (33) Diet cookies

12. (39) Baby foods, (129) Infant formulas, (230) Baby foods

***FRUITS***

1. (50) Raw, canned, frozen fruit without added sugar, (62) Unsweetened fruit juices,

(51) Canned sweetened fruit, (63) Sweetened fruit juices,

1. (52) Citrus fruits without added sugar, (53) Citrus fruits with added sugar, (60) Unsweetened citrus fruit juices, (61) Sweetened citrus fruit juices,
2. (54) Berries without added sugar, (55) Berries with added sugar
3. (56) Dried fruit, dried fruit cooked without added sugar, (57) Dried fruit cooked with added sugar
4. (58) Fruit desserts, (59) Fruit with added fat, (35) Fruit pies

***VEGETABLES***

1. (200) Raw and cooked without fat dark green vegetables, (204) Low sodium canned dark green vegetables, (202) Canned dark green vegetables with or without added fat, (201) Cooked with added fat dark green vegetables
2. (205) Raw and cooked without fat orange vegetables, (207) Canned orange vegetables with or without added fat**, (**209) Low sodium canned orange vegetables, (210) Cooked orange vegetables with sugar added, (206) Cooked with added fat orange vegetables, carrot juice
3. (211) Raw and cooked without fat starchy vegetables, (213) Canned starchy vegetables without added fat, (215) Low sodium canned starchy vegetables, (224) Canned vegetable combinations cooked with or without fat, (212) Cooked with added fat starchy vegetables, (214) Canned starchy vegetables with added fat, (216) French fried potatoes
4. (217) Raw and cooked without fat other vegetables, (219) Canned other vegetables without added fat, (221) Low sodium canned other vegetables, (222) Vegetable combinations cooked without fat, (218) Cooked with added fat other vegetables, (220) Canned other vegetables with added fat, (223) Vegetable combinations cooked with fat, (229) Vegetable salads with added fat, (228) Pickled vegetables, (226) Vegetable juices, (227) Low sodium vegetable juices, (232) Low sodium veg combinations

***MILK and MILK PRODUCTS***

1. (100) Regular milk
2. (101) Reduced fat milk, (102) Fat free milk
3. (112) Natural, regular cheese, (116) Low sodium cheese
4. (113) Natural, reduced fat and fat free cheese
5. (114) Processed, regular cheese
6. (115) Processed, reduced fat and fat free cheese
7. Regular dairy products:

(120) Regular cheese products (cottage, cream), (106) Regular cream, (108) Regular milk based beverages, (117) Regular cheese sauce, (122) Cheese based soups, (110) Regular yogurt (includes frozen)

1. Low fat dairy products:

(121) Lowfat or fat free cheese products (cottage, cream), (107) Reduced fat cream, (109) Reduced fat milk based beverages, (118) Low fat cheese sauce, (111) Lowfat yogurt (includes frozen)

1. Dairy desserts- regular: (123) Regular ice cream, (126) Regular pudding, (103) Condensed milk
2. Dairy desserts – low or ff desserts: (124) Light ice cream, (125) Fat free ice cream, (127) Reduced fat pudding, (162) nondairy frozen desserts

***MEATS***

1. (300) Lean red meats no added fat, (309) Veal, (311) Game
2. (301) Red meats with fat, (308) Lamb
3. (302) Chicken/poultry no added fat
4. (303) Chicken/poultry with added fat
5. (304) Fin fish no fat
6. (305) Fin fish with added fat
7. (306) Shellfish
8. Sandwich: (119) Cheese sandwich, (320) Beef/pork sandwiches, (321) Poultry sandwiches, (322) Fish sandwiches, (323) Bacon/sausage hot dog sandwiches, (324) Submarine sandwiches and luncheon meat sandwiches, (38) Sandwiches (croissant, turnover)
9. (307) Sausage/bacon/luncheon meats, (310) Organ meats
10. (312) Meat dishes, (325) Frozen meat meals, (329) Frozen veal meals,

(313) Chicken dishes, (327) Frozen chicken meals,

(314) Seafood dishes, (330) Frozen fish meals, (150) Hispanic dishes with meat, (160) Dumplings and egg rolls

1. Soups: (315) Mixed meats (stews, gumbo), (316) Beef/pork soups, (317) Poultry soups, (318) Seafood soups, (161) Grain-based soups with meat,(231)Vegetable soups
2. Diet Frozen meals: (326) meat, (328) chicken, (331) fish

***EGGS***

1. Egg dishes: (140) Egg dishes without fat, (141) Egg dishes with added fat, (142) Egg substitutes, (143) Egg sandwiches, (144) Frozen egg meals

***LEGUMES***

1. (250) Legumes prepared with fat, (251) Legumes prepared without fat, (252) Canned legumes prepared with fat, (253) Canned legumes prepared without fat, (254) Low sodium canned legumes, (255) Legume prepared dishes with meat, (256) Legume prepared dishes without meat, (257) Legume based soups, (258) Low sodium legume based soups, (104) Soy milk, (151) Hispanic dishes without meat

***MIXED DISHES***

1. Pizza: (152) Pizza with meat, (153) Pizza without meat

***BEVERAGES***

1. Coffee/ Tea: (80) Coffee, (83) Coffee substitutes, (84) Tea
2. Sweetened drinks: (89) Regular soft drinks, (81) Presweetened coffee, (85) Presweetened tea, (87) Fruit drinks
3. Diet drinks: (90) Diet soft drinks, (82) Coffee with low calorie sweeteners, (86) Tea with low calorie sweetener, (88) Low calories fruit drinks
4. (91) Alcoholic beverages

***SUGARS***

1. Sugar: (400) Added sugars, (402) Regular gelatin dessert
2. Sugar substitute: (401) Sugar substitutes, (403) Sugar free gelatin desserts, (405) Dietetic free/low calorie candy
3. (404) Candy

***FATS***

1. (420) Animal fats and salad dressings, (319) Meat gravy
2. (421) Vegetable fats and salad dressings
3. (422) Reduced calorie spreads and salad dressings

***NUTS***

1. (450) Nuts and nut butters, (451) Low sodium nuts and nut butters, (452) Peanut butter sandwiches

***OTHERS***

1. (470) Protein powders and meal replacements, (128) Milk based powders, Milk substitutes, nutritional beverage.
2. (480) Yeast
3. (490) Condiments
4. Water
