# Supplementary material for: Longitudinal change in the diet's monetary value is associated with its change in quality and micronutrient adequacy among urban adults
Source: PLoS One. 2018 Oct 12;13(10):e0204141. doi: 10.1371/journal.pone.0204141 (PMC6193582; doi:10.1371/journal.pone.0204141)
Supplement: S1 Table — (DOCX) [file pone.0204141.s003.docx]

**TABLE S1**. Baseline, follow-up and longitudinal annual rate of change in diet quality scores (HEI-2010 and MAR/NAR scores) by sex, race and poverty status, HANDLS 2004-2013^1,3^

|  | **Overall** | **Men** | **Women** | **P_sex_^2^** | **Whites** | **AA** | **P_race_^2^** | **Above Poverty** | **Below Poverty** | **P_pov_^2^** |
| --- | --- | --- | --- | --- | --- | --- | --- | --- | --- | --- |
|  | **N=1,466** | **N=597** | **N=869** |  | **N=589** | **N=877** |  | **N=861** | **N=605** |  |
|  | X ± SE | X ± SE | X ± SE |  | X ± SE | X ± SE |  | X ± SE | X ± SE |  |
| HEI-2010 total score |  |  |  |  |  |  |  |  |  |  |
| Baseline | 43.2±0.3 | 41.9±0.4 | 44.0±0.4 | <0.001 | 43.5±0.5 | 42.9±0.4 | 0.40 | 44.5±0.4 | 41.3±0.4 | <0.001 |
| Follow-up | 46.5±0.3 | 45.3±0.4 | 47.4±0.4 | <0.001 | 47.2±0.6 | 46.1±0.4 | 0.098 | 47.9±0.4 | 44.6±0.46 | <0.001 |
| Δ | +0.74±0.07 | +0.73±0.11 | +0.75±0.10 | 0.88 | +0.86±0.13 | +0.66±0.09 | 0.17 | +0.78±0.10 | +0.68±0.10 | 0.47 |
| Total vegetables |  |  |  |  |  |  |  |  |  |  |
| Baseline | 2.71±0.03 | 2.55±0.05 | 2.81±0.05 | <0.001 | 2.84±0.1 | 2.62±0.04 | 0.002 | 2.77±0.04 | 2.62±0.05 | 0.036 |
| Follow-up | 2.72±0.04 | 2.61±0.05 | 2.79±0.05 | 0.009 | 2.89±0.1 | 2.60±0.04 | <0.001 | 2.81±0.05 | 2.58±0.05 | 0.001 |
| Δ | +0.00±0.01 | +0.01±0.02 | +0.00±0.01 | 0.73 | +0.01±0.02 | -0.00±0.01 | 0.41 | +0.01±0.01 | -0.01±0.01 | 0.16 |
| Greens and beans |  |  |  |  |  |  |  |  |  |  |
| Baseline | 0.99±0.04 | 0.88±0.05 | 1.07±0.05 | 0.016 | 0.91±0.06 | 1.04±0.05 | 0.072 | 1.00±0.05 | 0.98±0.05 | 0.79 |
| Follow-up | 1.27±0.04 | 1.12±0.06 | 1.37±0.05 | 0.002 | 1.32±0.07 | 1.24±0.05 | 0.35 | 1.32±0.05 | 1.19±0.06 | 0.12 |
| Δ | +0.06±0.01 | +0.05±0.02 | +0.08±0.02 | 0.24 | **+0.10±0.02** | **+0.04±0.01** | **0.014** | +0.08±0.02 | +0.04±0.02 | 0.08 |
| Total fruit |  |  |  |  |  |  |  |  |  |  |
| Baseline | 1.63±0.04 | 1.53±0.07 | 1.71±0.06 | 0.035 | 1.70±0.07 | 1.59±0.05 | 0.22 | 1.84±0.06 | 1.35±0.06 | <0.001 |
| Follow-up | 1.87±0.05 | 1.65±0.07 | 2.02±0.06 | <0.001 | 1.80±0.07 | 1.92±0.04 | 0.21 | 1.97±0.06 | 1.73±0.07 | 0.009 |
| Δ | +0.04±0.01 | +0.02±0.02 | +0.06±0.02 | 0.11 | +0.02±0.02 | +0.06±0.01 | 0.074 | **+0.02±0.02** | **+0.07±0.02** | **0.030** |
| Whole fruit |  |  |  |  |  |  |  |  |  |  |
| Baseline | 1.25±0.04 | 1.11±0.06 | 1.35±0.06 | 0.006 | 1.56±0.08 | 1.05±0.05 | <0.001 | 1.45±0.06 | 0.97±0.06 | <0.001 |
| Follow-up | 1.57±0.05 | 1.34±0.07 | 1.72±0.06 | <0.001 | 1.80±0.08 | 1.41±0.06 | <0.001 | 1.73±0.06 | 1.34±0.07 | <0.001 |
| Δ | +0.07±0.01 | +0.05±0.02 | +0.08±0.02 | 0.19 | +0.06±0.02 | +0.08±0.01 | 0.33 | +0.07±0.02 | +0.07±0.02 | 0.77 |
| Whole grains |  |  |  |  |  |  |  |  |  |  |
| Baseline | 1.85±0.07 | 1.62±0.09 | +2.00±0.10 | 0.005 | 2.10±0.11 | 1.67±0.08 | 0.001 | 2.01±0.09 | 1.61±0.10 | 0.003 |
| Follow-up | 2.23±0.07 | 2.02±0.10 | +2.38±0.09 | 0.009 | 2.32±0.11 | 2.17±0.09 | 0.28 | 2.43±0.09 | 1.95±0.10 | <0.001 |
| Δ | +0.08±0.02 | +0.09±0.03 | +0.08±0.03 | 0.85 | +0.05±0.03 | +0.11±0.02 | 0.15 | +0.09±0.03 | +0.07±0.03 | 0.65 |
| Dairy |  |  |  |  |  |  |  |  |  |  |
| Baseline | 3.57±0.07 | 3.50±0.10 | +3.62±0.09 | 0.40 | 4.51±0.11 | 2.95±0.08 | <0.001 | 3.72±0.09 | 3.37±0.10 | 0.012 |
| Follow-up | 4.20±0.07 | 4.04±0.10 | +4.30±0.09 | 0.063 | 5.15±0.11 | 3.56±0.08 | <0.001 | 4.42±0.09 | 3.89±0.10 | <0.001 |
| Δ | +0.14±0.02 | +0.13±0.03 | +0.16±0.03 | 0.48 | +0.16±0.04 | +0.14±0.02 | 0.55 | +0.17±0.03 | 0.07±0.03 | 0.17 |
| Total protein foods |  |  |  |  |  |  |  |  |  |  |
| Baseline | 4.29±0.03 | 4.37±0.04 | 4.24±0.03 | 0.018 | 4.07±0.05 | 4.44±0.03 | <0.001 | 4.31±0.03 | 4.25±0.04 | 0.20 |
| Follow-up | 4.29±0.03 | 4.39±0.04 | 4.22±0.03 | 0.002 | 4.08±0.05 | 4.43±0.03 | <0.001 | 4.30±0.03 | 4.28±0.04 | 0.64 |
| Δ | +0.00±0.01 | +0.00±0.01 | +0.00±0.01 | 0.99 | +0.00±0.01 | +0.00±0.01 | 0.99 | -0.00±0.01 | +0.01±0.01 | 0.37 |
| Seafood and plant proteins |  |  |  |  |  |  |  |  |  |  |
| Baseline | 1.67±0.04 | 1.54±0.07 | 1.78±0.06 | 0.008 | 1.69±0.07 | 1.68±0.06 | 0.78 | 1.81±0.06 | 1.48±0.06 | <0.001 |
| Follow-up | 1.80±0.04 | 4.39±0.04 | 1.86±0.06 | 0.10 | 1.94±0.07 | 1.70±0.06 | 0.009 | 1.93±0.06 | 1.62±0.07 | <0.001 |
| Δ | +0.03±0.01 | +0.05±0.02 | +0.03±0.02 | 0.37 | **+0.07±0.02** | **+0.01±0.02** | **0.013** | +0.03±0.02 | +0.03±0.02 | 0.94 |
| Fatty acids |  |  |  |  |  |  |  |  |  |  |
| Baseline | 5.17±0.07 | 5.12±0.11 | 5.20±0.09 | 0.60 | 4.39±0.11 | 5.69±0.09 | <0.001 | 5.23±0.10 | 5.07±0.11 | 0.26 |
| Follow-up | 5.33±0.07 | 5.28±0.12 | 5.37±0.09 | 0.52 | 4.69±0.12 | 5.77±0.09 | <0.001 | 5.28±0.10 | 5.41±0.11 | 0.37 |
| Δ | +0.03±0.02 | +0.03±0.03 | +0.03±0.03 | 0.95 | +0.06±0.04 | +0.01±0.03 | 0.23 | +0.01±0.03 | +0.07±0.03 | 0.19 |
| Sodium |  |  |  |  |  |  |  |  |  |  |
| Baseline | 4.95±0.08 | 4.93±0.12 | 4.97±0.10 | 0.76 | 4.39±0.11 | 5.01±0.10 | 0.36 | 4.90±0.10 | 5.03±0.12 | 0.38 |
| Follow-up | 4.20±0.07 | 4.12±0.12 | 4.26±0.10 | 0.34 | 4.25±0.12 | 4.17±0.09 | 0.61 | 4.15±0.10 | 4.28±0.12 | 0.37 |
| Δ | -0.18±0.02 | -0.19±0.04 | -0.18±0.03 | 0.78 | -0.17±0.04 | -0.19±0.03 | 0.55 | -0.19±0.03 | -0.17±0.03 | 0.56 |
| Refined grains |  |  |  |  |  |  |  |  |  |  |
| Baseline | 6.23±0.07 | 6.11±0.12 | 6.33±0.09 | 0.16 | 5.89±0.12 | 6.47±0.09 | <0.001 | 6.21±0.10 | 6.29±0.11 | 0.58 |
| Follow-up | 6.61±0.07 | 6.61±0.11 | 6.61±0.09 | 0.94 | 6.31±0.11 | 6.82±0.09 | 0.0003 | 6.60±0.09 | 6.63±0.11 | 0.80 |
| Δ | +0.09±0.02 | +0.10±0.04 | +0.08±0.03 | 0.59 | +0.11±0.04 | +0.08±0.03 | 0.51 | +0.09±0.03 | +0.08±0.03 | 0.73 |
| Empty calories |  |  |  |  |  |  |  |  |  |  |
| Baseline | 8.83±0.14 | 8.61±0.22 | 8.97±0.18 | 0.20 | 9.00±0.23 | 8.73±0.17 | 0.37 | 9.21±0.19 | 8.28±0.21 | 0.001 |
| Follow-up | 10.44±0.14 | 10.38±0.21 | 10.48±0.18 | 0.71 | 10.64±0.23 | 10.31±0.17 | 0.24 | 10.95±0.18 | 9.70±0.21 | <0.001 |
| Δ | +0.35±0.04 | +0.39±0.06 | +0.33±0.04 | 0.45 | 0.39±0.06 | +0.33±0.04 | 0.46 | +0.40±0.05 | +0.29±0.05 | 0.16 |
| MAR |  |  |  |  |  |  |  |  |  |  |
| Baseline | 73.3±0.4 | 77.1±0.6 | 70.7±0.5 | <0.001 | 75.7±0.6 | 71.6±0.5 | <0.001 | 73.7±0.5 | 72.7±0.6 | 0.25 |
| Follow-up | 75.4±0.4 | 78.1±0.5 | 73.6±0.5 | <0.001 | 77.3±0.6 | 74.1±0.5 | <0.001 | 76.6±0.5 | 73.7±0.6 | <0.001 |
| Δ | 0.48±0.10 | **+0.23±0.13** | **0.65±0.13** | **0.032** | +0.36±0.16 | +0.56±0.12 | 0.30 | **+0.68±0.13** | **+0.20±0.13** | **0.014** |
| Vitamin A, NAR |  |  |  |  |  |  |  |  |  |  |
| Baseline | 53.2±0.8 | 56.3±1.2 | 51.2±1.0 | 0.001 | 58.3±1.2 | 49.9±1.0 | <0.001 | 53.7±1.0 | 52.6±1.2 | 0.49 |
| Follow-up | 63.2±0.8 | 60.5±1.2 | 65.0±1.0 | 0.004 | 67.6±1.2 | 60.2±1.0 | <0.001 | 65.6±1.0 | 59.7±1.2 | <0.001 |
| Δ | +2.37±0.22 | **+0.99±0.35** | **+3.33±0.28** | **<0.001** | +2.46±0.38 | +2.32±0.27 | 0.76 | **+2.96±0.30** | **+1.54±0.31** | **0.002** |
| Vitamin C, NAR |  |  |  |  |  |  |  |  |  |  |
| Baseline | 53.8±0.9 | 55.4±1.5 | 52.7±1.2 | 0.14 | 52.2±1.5 | 54.9±1.2 | 0.15 | 56.3±1.2 | 50.2±1.4 | 0.001 |
| Follow-up | 63.0±0.9 | 59.1±1.5 | 65.7±1.2 | <0.001 | 59.5±1.5 | 65.4±1.2 | 0.002 | 64.9±1.2 | 60.4±1.5 | 0.016 |
| Δ | 1.86±0.26 | **+0.58±0.42** | **+2.73±0.33** | **<0.001** | +1.66±0.43 | +2.00±0.33 | 0.53 | +1.80±0.36 | +1.94±0.38 | 0.79 |
| Vitamin D, NAR |  |  |  |  |  |  |  |  |  |  |
| Baseline | 23.7±0.5 | 27.1±0.9 | 21.4±0.7 | <0.001 | 25.5±0.9 | 22.5±0.66 | 0.006 | 23.6±0.7 | 23.8±0.8 | 0.84 |
| Follow-up | 19.0±0.5 | 23.5±0.9 | 16.0±0.6 | <0.001 | 20.8±0.9 | 17.9±0.6 | 0.007 | 19.8±0.7 | 17.9±0.8 | 0.064 |
| Δ | -0.87±0.17 | -0.68±0.28 | -1.00±0.22 | 0.37 | -1.08±0.25 | -0.73±0.24 | 0.32 | -0.80±0.21 | -0.97±0.29 | 0.63 |
| Vitamin E, NAR |  |  |  |  |  |  |  |  |  |  |
| Baseline | 42.2±0.6 | 46.5±1.0 | 39.2±0.8 | <0.001 | 44.6±1.0 | 40.5±0.77 | 0.001 | 42.9±0.8 | 41.2±0.9 | 0.17 |
| Follow-up | 46.8±0.6 | 51.5±1.0 | 43.5±0.8 | <0.001 | 48.7±1.0 | 45.5±0.8 | 0.012 | 48.7±0.8 | 44.0±0.9 | <0.001 |
| Δ | +1.06±0.17 | +1.14±0.27 | +1.00±0.21 | 0.67 | +1.00±0.29 | +2.32±0.27 | 0.77 | **+1.40±0.24** | **+0.57±0.23** | **0.015** |
| Vitamin B-6, NAR |  |  |  |  |  |  |  |  |  |  |
| Baseline | 85.9±0.5 | 89.3±0.8 | 83.6±0.7 | <0.001 | 87.1±0.8 | 85.1±0.7 | 0.068 | 85.9±0.7 | 85.9±0.8 | 0.90 |
| Follow-up | 87.5±0.5 | 90.8±0.7 | 85.2±0.7 | <0.001 | 87.0±0.9 | 87.8±0.6 | 0.47 | 88.3±0.7 | 86.3±0.8 | 0.062 |
| Δ | +0.38±0.15 | +0.38±0.19 | +0.38±0.21 | 0.99 | **-0.01±0.25** | **+0.64±0.18** | **0.031** | +0.56±0.20 | +0.12±0.22 | 0.14 |
| Vitamin B-12, NAR |  |  |  |  |  |  |  |  |  |  |
| Baseline | 91.5±0.5 | 94.6±0.6 | 89.3±0.7 | <0.001 | 93.0±0.7 | 90.4±0.7 | 0.013 | 91.1±0.7 | 92.0±0.7 | 0.34 |
| Follow-up | 91.8±0.5 | 94.2±0.6 | 90.2±0.7 | <0.001 | 93.6±0.7 | 90.7±0.7 | 0.003 | 92.4±0.6 | 91.1±0.8 | 0.19 |
| Δ | +0.12±0.14 | -0.08±0.19 | 0.27±0.20 | 0.22 | +0.17±0.21 | +0.09±0.19 | 0.77 | **+0.35±0.19** | **-0.21±0.20** | **0.048** |
| Thiamin, NAR |  |  |  |  |  |  |  |  |  |  |
| Baseline | 87.8±0.5 | 90.1±0.7 | 86.2±0.7 | <0.001 | 92.8±0.6 | 85.6±0.7 | <0.001 | 88.1±0.6 | 87.4±0.8 | 0.49 |
| Follow-up | 90.5±0.5 | 93.1±0.6 | 88.6±0.6 | <0.001 | 91.1±0.7 | 88.9±0.6 | <0.001 | 91.0±0.6 | 89.6±0.7 | 0.13 |
| Δ | +0.54±0.13 | +0.71±0.19 | +0.43±0.19 | 0.31 | +0.32±0.21 | 0.70±0.18 | 0.17 | +0.63±0.18 | +0.42±0.21 | 0.45 |
| Riboflavin, NAR |  |  |  |  |  |  |  |  |  |  |
| Baseline | 94.0±0.4 | 95.0±0.5 | 93.2±0.5 | 0.015 | 96.2±0.5 | 92.5±0.5 | <0.001 | 93.9±0.5 | 94.1±0.6 | 0.71 |
| Follow-up | 94.1±0.4 | 95.1±0.5 | 93.4±0.5 | 0.017 | 96.7±0.4 | 92.4±0.5 | <0.001 | 94.9±0.4 | 93.0±0.6 | 0.011 |
| Δ | +0.03±0.10 | +0.02±0.15 | +0.04±0.14 | 0.92 | +0.11±0.14 | -0.02±0.14 | 0.52 | **+0.21±0.13** | **-0.22±0.15** | **0.035** |
| Niacin, NAR |  |  |  |  |  |  |  |  |  |  |
| Baseline | 91.7±0.4 | 95.7±0.5 | 89.0±0.6 | <0.001 | 92.6±0.6 | 91.1±0.6 | 0.076 | 91.9±0.5 | 91.4±0.7 | 0.56 |
| Follow-up | 95.1±0.3 | 96.8±0.4 | 93.9±0.5 | <0.001 | 94.9±0.5 | 95.2±0.4 | 0.69 | 95.4±0.4 | 94.7±0.5 | 0.29 |
| Δ | +0.72±0.11 | **+0.29±0.15** | **+1.02±0.16** | **0.002** | +0.48±0.20 | +0.89±0.14 | 0.08 | +0.80±0.16 | +0.61±0.17 | 0.40 |
| Folate, NAR |  |  |  |  |  |  |  |  |  |  |
| Baseline | 72.8±0.7 | 77.8±1.0 | 69.3±0.9 | <0.001 | 77.3±1.0 | 69.7±0.8 | <0.001 | 73.1±0.9 | 72.3±1.0 | 0.56 |
| Follow-up | 75.7±0.6 | 81.3±0.9 | 71.7±0.8 | <0.001 | 80.5±0.9 | 72.4±0.8 | <0.001 | 77.8±0.8 | 72.6±1.0 | <0.001 |
| Δ | +0.65±0.17 | +0.80±0.24 | +0.54±0.23 | 0.46 | +0.71±0.28 | +0.60±0.21 | 0.75 | **+1.09±0.23** | **+0.02±0.24** | **0.002** |
| Iron, NAR |  |  |  |  |  |  |  |  |  |  |
| Baseline | 78.5±0.7 | 97.5±0.4 | 65.4±0.8 | <0.001 | 80.3±1.0 | 77.1±0.9 | 0.020 | 79.0±0.9 | 77.3±1.00 | 0.35 |
| Follow-up | 89.6±0.5 | 98.1±0.3 | 83.8±0.8 | <0.001 | 90.2±0.8 | 89.3±0.7 | 0.35 | 90.3±0.6 | 88.7±0.83 | 0.14 |
| Δ | +2.48±0.15 | **+0.13±0.11** | **+4.10±0.23** | **<0.001** | +2.38±0.25 | +2.56±0.19 | 0.57 | +2.64±0.21 | +2.27±0.22 | 0.23 |
| Copper, NAR |  |  |  |  |  |  |  |  |  |  |
| Baseline | 90.6±0.4 | 93.8±0.5 | 88.5±0.6 | 0.006 | 91.4±0.7 | 90.1±0.6 | 0.16 | 91.1±0.5 | 90.00±0.68 | 0.21 |
| Follow-up | 90.2±0.4 | 93.2±0.5 | 88.1±0.6 | <0.001 | 91.3±0.7 | 89.4±0.6 | 0.033 | 91.2±0.5 | 88.7±0.83 | 0.005 |
| Δ | -0.11±0.11 | -0.08±0.16 | -0.12±0.17 | 0.86 | -0.08±0.20 | -0.13±0.15 | 0.85 | +0.03±0.14 | -0.31±0.17 | 0.17 |
| Zinc, NAR |  |  |  |  |  |  |  |  |  |  |
| Baseline | 86.1±0.5 | 85.6±0.8 | 86.4±0.7 | 0.44 | 84.3±0.7 | 88.7±0.8 | <0.001 | 85.5±0.7 | 86.9±0.8 | 0.18 |
| Follow-up | 64.5±0.6 | 68.7±1.0 | 61.6±0.8 | <0.001 | 62.4±0.8 | 67.6±1.0 | <0.001 | 65.7±0.8 | 62.8±1.0 | 0.021 |
| Δ | -4.87±0.17 | **-3.77±0.26** | **-5.63±0.22** | **<0.001** | -4.64±0.21 | -5.20±0.30 | 0.11 | -4.75±0.24 | -5.04±0.24 | 0.42 |
| Calcium, NAR |  |  |  |  |  |  |  |  |  |  |
| Baseline | 62.3±0.7 | 70.0±1.1 | 57.0±0.9 | <0.001 | 68.6±1.1 | 58.1±0.9 | <0.001 | 63.2±0.9 | 61.0±1.1 | 0.11 |
| Follow-up | 71.1±0.7 | 79.2±1.0 | 65.6±0.9 | <0.001 | 76.6±1.0 | 67.5±0.9 | <0.001 | 73.0±0.9 | 68.5±1.0 | 0.001 |
| Δ | +2.07±0.18 | +2.14±0.27 | +2.02±0.24 | 0.73 | +1.95±0.28 | +2.14±0.23 | 0.58 | **+2.38±0.24** | **+1.62±0.26** | **0.035** |
| Magnesium, NAR |  |  |  |  |  |  |  |  |  |  |
| Baseline | 63.2±0.6 | 61.1±0.9 | 64.6±0.8 | <0.001 | 68.4±1.0 | 59.6±0.7 | 0.003 | 64.2±0.8 | 61.8±0.9 | 0.051 |
| Follow-up | 67.3±0.6 | 65.4±0.9 | 68.6±0.8 | 0.006 | 71.5±0.9 | 64.5±0.7 | <0.001 | 69.5±0.8 | 64.2±0.9 | <0.001 |
| Δ | +0.90±0.15 | +0.91±0.23 | +0.90±0.19 | 0.98 | +0.61±0.26 | 1.10±0.18 | 0.11 | **+1.19±0.21** | **+0.49±0.21** | **0.022** |
| Phosphorus, NAR |  |  |  |  |  |  |  |  |  |  |
| Baseline | 95.4±0.3 | 97.6±0.4 | 93.9±0.5 | <0.001 | 96.6±0.4 | 94.7±0.4 | <0.001 | 95.6±0.4 | 95.2±0.5 | 0.59 |
| Follow-up | 97.0±0.3 | 98.6±0.3 | 95.9±0.4 | <0.001 | 97.6±0.4 | 96.5±0.4 | 0.048 | 97.1±0.3 | 94.7±0.4 | 0.45 |
| Δ | +0.32±0.08 | +0.21±0.10 | +0.40±0.13 | 0.29 | +0.22±0.13 | +0.39±0.11 | 0.33 | +0.36±0.11 | +0.27±0.12 | 0.60 |
|  |  |  |  |  |  |  |  |  |  |  |

*Abbreviations:* Δ=Annual rate of change; HEI-2010= Healthy Eating Index, 2010 version; HANDLS=Healthy Aging in Neighborhood of Diversity across the Lifespan; MAR=Mean Adequacy Ratio, MVD=Monetary value of the diet, NAR=Nutrient Adequacy Ratio; SE=Standard Error.

^1^ Values are means ± standard errors for baseline, follow-up and annual rates of change (Δ) of diet quality indices, total scores and components. Components of the mean adequacy score (MAR) and the individual nutrient adequacy scores for each micronutrient (NAR). Both MAR and NAR can range from 0 to 100. Total score for HEI-2010 can range from 0 to 100, but component scores vary in possible ranges (e.g. 0-20 for empty calories vs. 0-5 for total fruit).

^2^ 2-sided P-value associated with an independent samples *t*-test for comparing means across sex, race or poverty status.

^3^Researchers own analyses and calculations based in part on data reported by Nielsen through its Homescan Service for the food and beverage categories for the years 2004-2013, for the US market  Nielsen data is licensed from The Nielsen Company, 2016  The conclusions drawn from the Nielsen data are those of the Researchers and do not reflect the views of Nielsen.  Nielsen is not responsible for and was not involved in analyzing and preparing the results reported herein.
