# Supplementary material for: Longitudinal change in the diet's monetary value is associated with its change in quality and micronutrient adequacy among urban adults
Source: PLoS One. 2018 Oct 12;13(10):e0204141. doi: 10.1371/journal.pone.0204141 (PMC6193582; doi:10.1371/journal.pone.0204141)
Supplement: S3 Fig — (PPTX) [file pone.0204141.s006.pptx]

## Slide 1
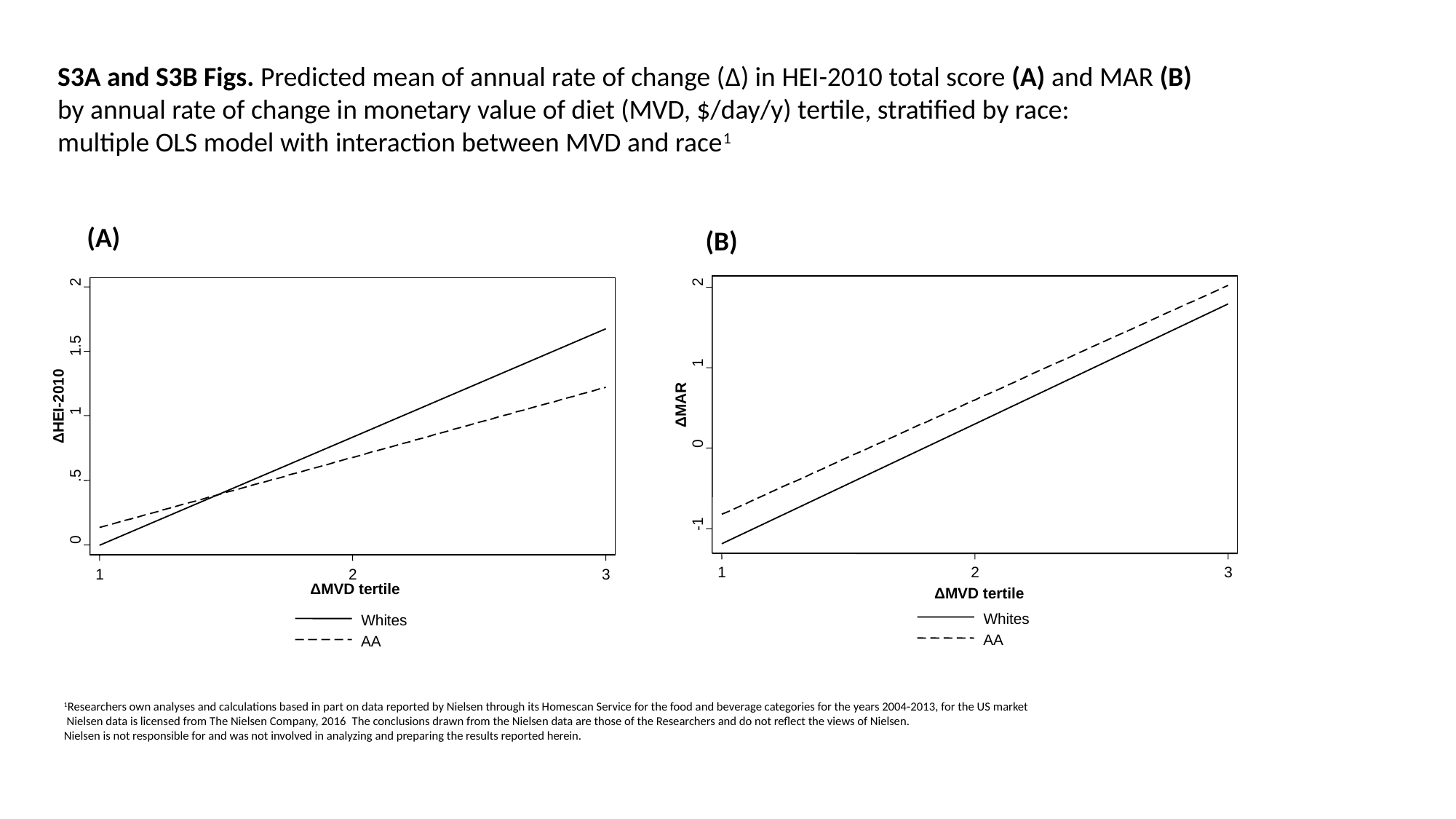

S3A and S3B Figs. Predicted mean of annual rate of change (Δ) in HEI-2010 total score (A) and MAR (B)
by annual rate of change in monetary value of diet (MVD, $/day/y) tertile, stratified by race:
multiple OLS model with interaction between MVD and race1
(A)
(B)
2
1
ΔMAR
0
-1
1
2
3
ΔMVD tertile
Whites
AA
2
1.5
ΔHEI-2010
1
.5
0
1
2
3
ΔMVD tertile
Whites
AA
1Researchers own analyses and calculations based in part on data reported by Nielsen through its Homescan Service for the food and beverage categories for the years 2004-2013, for the US market
 Nielsen data is licensed from The Nielsen Company, 2016  The conclusions drawn from the Nielsen data are those of the Researchers and do not reflect the views of Nielsen.
Nielsen is not responsible for and was not involved in analyzing and preparing the results reported herein.
